# High-throughput screening of inorganic compounds for the discovery of novel dielectric and optical materials

**DOI:** 10.1038/sdata.2016.134

**Published:** 2017-01-31

**Authors:** Ioannis Petousis, David Mrdjenovich, Eric Ballouz, Miao Liu, Donald Winston, Wei Chen, Tanja Graf, Thomas D. Schladt, Kristin A. Persson, Fritz B. Prinz

**Affiliations:** 1 Department of Materials Science and Engineering, Stanford University, Stanford, California 94305, USA; 2 Department of Materials Science and Engineering, Hearst Mining Memorial Building, Berkeley, California 94720, USA; 3 Department of Mechanical Engineering, Stanford University, Stanford, California 94305, USA; 4 Lawrence Berkeley National Laboratory, 1 Cyclotron Rd, Berkeley, California 94720, USA; 5 Department of Mechanical, Materials and Aerospace Engineering, Illinois Institute of Technology, Chicago, Illinois 60616, USA; 6 Volkswagen Group Research, Berliner Ring 2, Wolfsburg 38840, Germany

**Keywords:** Electronic structure, Electronic devices, Density functional theory, Electronic and spintronic devices

## Abstract

Dielectrics are an important class of materials that are ubiquitous in modern electronic applications. Even though their properties are important for the performance of devices, the number of compounds with known dielectric constant is on the order of a few hundred. Here, we use Density Functional Perturbation Theory as a way to screen for the dielectric constant and refractive index of materials in a fast and computationally efficient way. Our results constitute the largest dielectric tensors database to date, containing 1,056 compounds. Details regarding the computational methodology and technical validation are presented along with the format of our publicly available data. In addition, we integrate our dataset with the Materials Project allowing users easy access to material properties. Finally, we explain how our dataset and calculation methodology can be used in the search for novel dielectric compounds.

## Background & Summary

Dielectric materials are an important component for a plethora of applications in modern electronics, such as Dynamic Random Access Memory (DRAM), flash memory, the Central Processing Unit (CPU), Light Emitting Diodes (LED) and photovoltaics. While high-k dielectrics enable more charge to be stored per unit volume, thus improving performance and driving device size down, low-k materials limit cross-communication, thus enabling devices to be packed closer together. As a result, new dielectric materials with tailored properties are essential for more efficient and better performing electronics as well as miniaturization. Furthermore, with the increasing use of electronics and electric motors in engineering applications, dielectric materials are starting to play a key role in industries such as, automotive, shipping and aerospace. Their specific requirements, however, are quite different to those of consumer electronics, typically requiring longer life as well as greater resistance to mechanical stress and temperature fluctuations.

As a result, there is a need for novel dielectric materials with properties suitable for a range of applications across different industries. However, the number of compounds with known dielectric constant is currently on the order of a few hundred, which drastically limits the options available to the design engineer. The number of inorganic compounds is on the order of 30–50,000 (refs 1–3) hence, there exist tens of thousands of compounds for which the dielectric response remains unknown. Given the sheer size of the chemical compound space, attempting to experimentally search for new dielectrics is not practical considering the time required for synthesis and measurement. On the other hand, Density Functional Perturbation Theory (DFPT) provides a relatively fast and inexpensive method to build a comprehensive dataset from which to derive structure/chemistry—dielectric property correlations and scan for interesting compounds.

**Figure 1:**
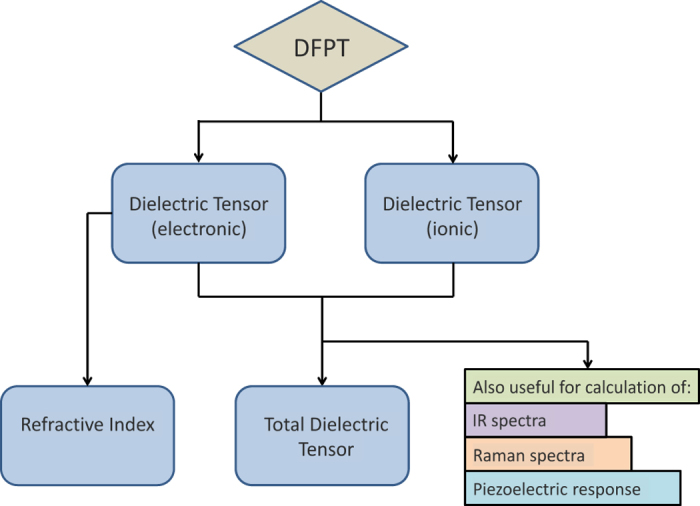
Illustration summarizing the quantities calculated in this work and their relation to other, fundamental, material properties.

The dielectric tensor of a material relates the electric field within the material to that applied externally and is comprised of the electronic as well as ionic contributions. In addition to its importance in defining low- and high-k materials, the dielectric tensor is also useful in the calculation of other material properties (Fig. 1). For example, as demonstrated by Petousis *et al.*^4^, it is possible to estimate the refractive index, *n*, of compounds at optical frequencies with ~6% deviation from experiments using static DFPT calculations. Furthermore, the ionic and electronic components of the dielectric tensor can be used to predict both the Infrared (IR) and Raman spectra of compounds. Along with the elastic^5^ and piezoelectric^6^ tensors, the dielectric tensor provides all the information necessary for the solution of the constitutive equations in applications where electric and mechanical stresses are coupled.

Previous studies range from single-compound investigations to high-throughput screening of polymers. More specifically, one high-throughput study^7^ was related to a specific system (Zr_*x*_Si_1−*x*_O_2_) and another reported the average dielectric constant for a few tens of inorganic compounds^8^. There have also been high-throughput studies on dielectrics, specific to organic polymers^9^. Experimental databases also exist but the total number of compounds listed is on the order of a few hundred.

In this work, we use the methodology established in Petousis *et al.*^4^ to generate the largest database of dielectric tensors to date consisting of 1,056 inorganic ordered compounds. Specifically, we report the full dielectric tensor for the total response as well as the electronic and ionic contributions. We also provide an estimate for the refractive index at optical wavelengths and far from resonance. It is worth noting that some of the listed compounds are hypothetical, created *in silico* by e.g., structure-prediction algorithms^10^ and have, to our knowledge, not been synthesized yet. As our present work focuses on compound screening, we did not deliberately single out any specific chemical compositions and/or structures for calculation. Our results are integrated in the Materials Project^11^ which is an open database and aims at employing high-throughput methods to predicting material properties for discovery and design.

## Methods

### Theory and definitions

Formally, the dielectric tensor *ε* relates the externally applied electric field to the field within the material and can be defined as:

(1)Ei=∑jεij−1E0j

where *E* is the electric field inside the material and *E*_0_ is the externally applied electric field. the indices *i*, *j* refer to the direction in space and take the values: {1, 2, 3}. The dielectric tensor can be split in the ionic (*ϵ*_0_) and electronic (*ϵ*_∞_) contributions:

(2)εij=εij0+εij∞

Here, we consider only the response of non-zero band gap materials to time-invariant fields. In the hypothetical case that a material does not respond at all to the external field, $\varepsilon_{ij}^{\infty }$ would be equal to the identity tensor and $\varepsilon_{ij}^{0}$ would be zero. In fact, materials with zero ionic contribution do exist. In general, for $\varepsilon_{ij}^{0}$ to be non-zero, compounds need to have at least 2 atoms per primitive cell, each having a different atomic charge.

The dielectric tensor is symmetric and respects all the symmetry operations of the corresponding point group. This limits the number of independent elements in the tensor to a minimum of 1 and a maximum of 6 depending on the crystal symmetry (Table 1).

The dielectric response calculated herein corresponds to that of a single crystal. In polycrystalline samples, grains are oriented randomly and hence, the actual response will be different. Nevertheless, the upper and lower bounds of the polycrystalline dielectric constant have proven to be^12^:

(3)31/λ1+1/λ2+1/λ3<εpoly<λ1+λ2+λ33

where *λ*_1_, *λ*_2_, *λ*_3_ are the eigenvalues of the single crystal dielectric tensor. Of course, the above inequality takes into account only the different orientation of the grains and ignores effects due to e.g., impurities or other kinds of defects. For the sake of simplicity, here we estimate the polycrystalline dielectric constant using the simple average, i.e., we define:

(4)εpoly≡λ1+λ2+λ33

Generally, the dielectric response varies with the frequency of the applied external field however here, we consider the static response (i.e., the response at constant electric fields or the long wavelength limit). Since the ionic contribution vanishes at high frequencies, our results can be used to obtain an estimate of the refractive index, *n*, at optical frequencies and far from resonance effects using the well known formula^4^:

(5)n=εpoly∞

where $\varepsilon_{poly}^{\infty }$ is the average of the eigenvalues of the electronic contribution to the dielectric tensor. It should be noted that equation (5) assumes the material is non-magnetic.

### Computational workflow

**Figure 2:**
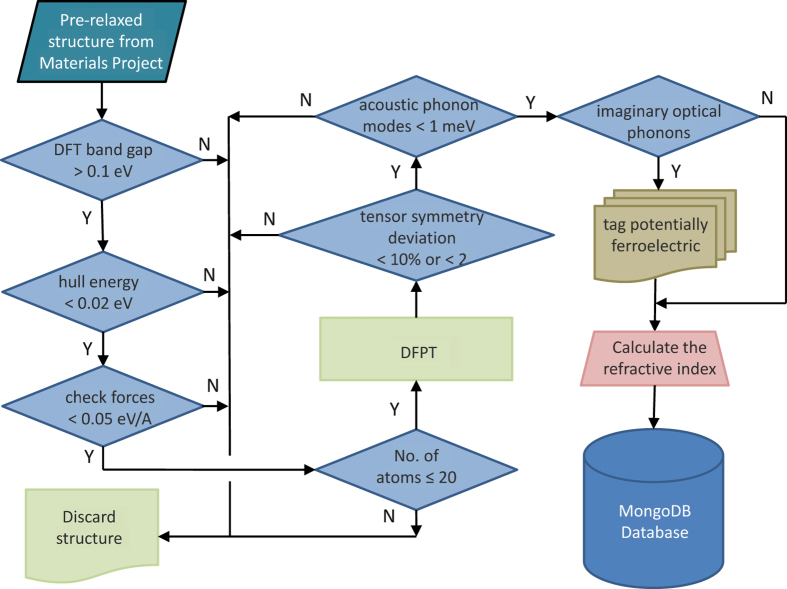
The workflow for calculating the dielectric tensor.

The workflow for calculating the dielectric constant is similar to the one used and extensively benchmarked against experimental data, by Petousis *et al.*^4^ (Fig. 2). All structures were downloaded from the Materials Project database^11,13,14^. To ensure a good starting set of materials (e.g., well-relaxed, stable structures), we apply the following 3 selection criteria: 1) the DFT band gap should be greater than 0.1 eV, 2) the hull energy in the phase diagram should be less than 0.02 eV and 3) the interatomic forces of the starting structure should be less than 0.05 eV/Å. It should be noted here that since we are using perturbation theory, our structure should ideally be as close to the ground state as possible. However, in practice^4^ we found that a threshold value of 0.05 eV/Å for the interatomic forces leads to acceptable errors for our screening methodology. For computational efficiency we, at this point, limit the set of calculated compounds to those with ≤20 atoms per supercell. After the DFPT calculation, the validity of the calculation is checked by ensuring the energy of the acoustic phonon modes at the Gamma point is less than 1 meV and that the dielectric tensor respects the point group symmetry operations with an error less or equal to 10% (relative) or 2 (absolute). The latter was in practice implemented by applying the symmetry operation to the tensor and ensuring that no tensor element changed by more than 10% or 2 with respect to the mean value of the original tensor element and of the tensor element after the symmetry operation was applied.’ Furthermore, if we find imaginary optical phonon modes at the Gamma point, we tag those compounds as potentially ferroelectric.

For the DFPT calculations we used the Vienna Ab-Initio Simulation Package^15–18^ (VASP version 5.3.4) combined with the Generalized Gradient Approximation GGA/PBE^19,20^+U^21,22^ exchange-correlation functional and Projector Augmented Wave pseudopotentials^23,24^. The U values are energy corrections that address the spurious self-interaction energy introduced by GGA. Here, we used U values for *d* orbitals only that were fitted to experimental binary formation enthalpies using Wang *et al.*’^25^ method. The full list of U values used, can be found in ref. 22. The k-point density was set at 3,000 per reciprocal atom and the plane wave energy cut-off at 600 eV (ref. 4). For detailed information on the calculation of the dielectric tensor within the DFPT framework we refer to Baroni *et al.*^26,27^ and Gonze & Lee^28^.

### Code availability

The DFPT calculations in this work were performed using the proprietary code VASP. The pre-and post-processing of the simulations was achieved using pymatgen^13^ and FireWorks^29^. Pymatgen^13^ is open-source software under the Massachusetts Institute of Technology (MIT) license. The workflow in Fig. 2 was implemented in FireWorks^29^ which is publicly available under a modified GNU General Public License.

## Data Records

The calculated dielectric tensors and refractive indices are available on the Materials Project^13^ website (www.materialsproject.org) and can be downloaded using the Materials Project API^14^. On the website, it is also possible to query for compounds with a certain dielectric response and refractive index by applying the appropriate filters on the search engine. Additionally, the Materials Project website provides information about the simulation parameters, crystal structure and other properties. The results are also available in the form of a JSON file that can be downloaded directly from the Dryad repository (Data Citation 1).

### File format

The data for each of the calculated compounds are stored in a list and are provided as a JSON file. For each compound, there are key values, such as ‘e_electronic’ and ‘e_ionic’, that point to the appropriate property (Table 2). The key ‘meta’ contains all the appropriate metadata and has its own keys which are one level down in hierarchy. The metadata keys are presented in Table 3.

### Graphical representation of results

**Figure 3:**
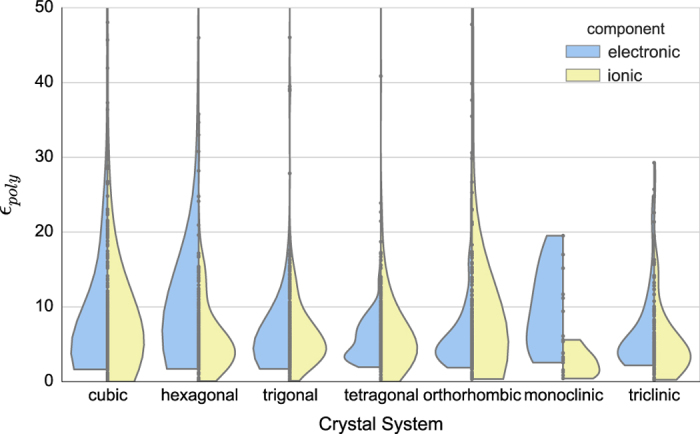
The polycrystalline estimate of the dielectric constant for the different crystal systems. The violin outlines are the Gaussian kernel density estimates of the data points that appear in the middle and are calculated using $f(x)=\frac{1}{nh}\sum_{i=1}^{n}K\left(\frac{x-x_{i}}{h}\right)$ where K is the Gaussian kernel and *h* is a smoothing parameter (estimated here using Scott's normal reference rule^30^). The left (blue) refers to the electronic component while the right (yellow), to the ionic. The total number of compounds in each crystal system was: 236, 132, 254, 183, 166, 11 and 74 (for cubic, hexagonal, trigonal, tetragonal, orthogonal, monoclinic and triclinic respectively).

In Fig. 3, we show a violin plot of the electronic and ionic contribution components of the dielectric constant for all calculated compounds, grouped according to the crystal system. Firstly, the plot shows that, as discussed above, the ionic contribution can be zero in contrast to its electronic counterpart. Furthermore, we observe the distribution of $\varepsilon_{poly}^{0}$ to be similar and relatively larger for cubic and orthorhombic crystals. However, monoclinic and triclinic crystals show lower values for the ionic component. This could be due to the lower level of symmetry and hence, the lack of phonon contributions to $\varepsilon_{ij}^{0}$.

**Figure 4:**
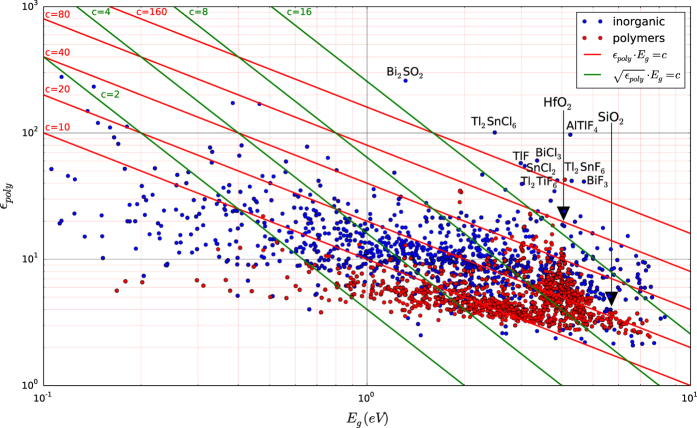
The polycrystalline estimate of the dielectric constant $\left(\varepsilon_{poly}\right)$. The x-axis represents the DFT-GGA+U band gap values (*E*_*g*_) obtained from the Materials Project website. There is a total of 1,056 data points for inorganic compounds. The red data points refer to values for polymers calculated by Huan *et al.*^31^ Red and green lines represent the figures of merit $\left(\varepsilon_{poly}\cdot E_{g}\right)$ and $\left(\sqrt{\varepsilon_{poly}}\cdot E_{g}\right)$ respectively. The formulas of promising compounds with $\varepsilon_{poly}\cdot E_{g}>16$ **and** $\sqrt{\varepsilon_{poly}}\cdot E_{g}>160$, are shown on the graph. HfO_2_, SiO_2_ and polyethylene are also shown for information.

**Figure 5:**
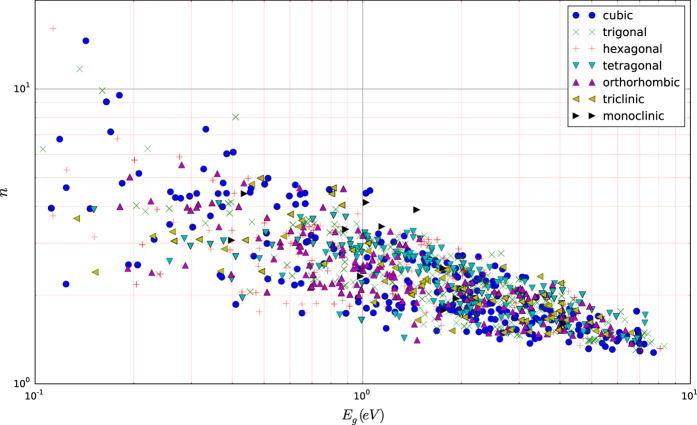
The polycrystalline estimate of the refractive index versus band gap for the different crystal systems. There appears to be no trend between the refractive index/band gap inverse relationship and the crystal system the material belongs to.

**Figure 6:**
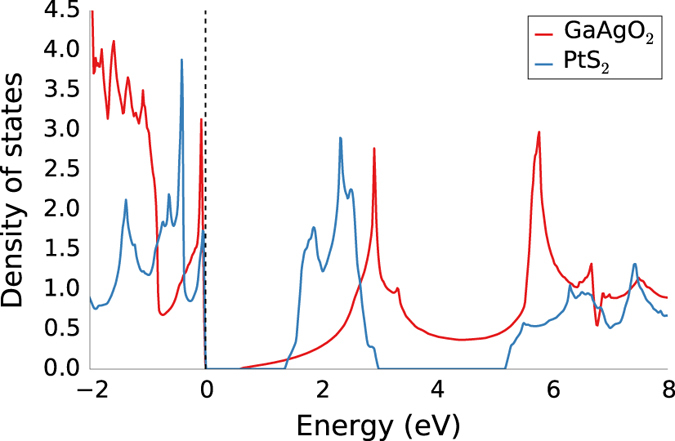
The density of states for PtS_2_ and GaAgO_2_. PtS_2_ has a higher electronic dielectric constant than GaAgO_2_, even though its band gap is larger.

We have also plotted the results versus the band gap predicted by DFT-GGA+U (we note that DFT-GGA+U has the tendency to systematically underestimate band gaps). Figures 4 and 5 show the variation of $\varepsilon_{total}$ and *n* with band gap, respectively. Additionally, in Fig. 4 we plotted the dielectric constant of polymers calculated by Sharma *et al.*^9^. Both figures demonstrate the inverse dependence of the dielectric constant with the band gap. In fact, the trend is more pronounced for the refractive index since $n=\sqrt{\varepsilon_{poly}^{\infty }}$ and hence, phonon contributions are excluded. The inverse relationship should be expected because if one considers 1st order perturbation theory, the electronic susceptibility depends inversely on the energy difference of the transition states (the latter increases, on average, with increasing band gap). However, we also observe that for a given band gap, the dielectric constant can take a range of values hinting that other aspects of the band structure are also important. Indeed as expected, compounds with a large number of states close to or at the valence/conduction bands maxima/minima have a relatively larger number of low energy transition states and hence, a relatively higher electronic dielectric constant. This is demonstrated in Fig. 6 where PtS_2_ ($\varepsilon_{poly}^{\infty }\approx 10$) has a larger dielectric constant than GaAgO_2_ ($\varepsilon_{poly}^{\infty }\approx 6$) even though its band gap is also larger.

### High-k dielectrics

In Fig. 4 we superimposed the lines $\varepsilon_{poly}\cdot E_{g}=c$ and $\sqrt{\varepsilon_{poly}}\cdot E_{g}=c$ (where *E*_*g*_ represents the band gap of the material and *c* is a constant). These quantities are proxies to the figures of merit for current leakage^8^ and energy storage^9^ of a capacitor respectively. Since for high-k dielectrics, both high $\varepsilon_{poly}\cdot E_{g}$ and $\sqrt{\varepsilon_{poly}}\cdot E_{g}$ are desired in order to limit leakage and maximize energy storage in applications, we identified the best performing compounds out of the ones calculated and highlighted them in Fig. 4. Thus, the design of new and better performing dielectric materials effectively becomes a battle against the inverse relationship between *E*_*g*_ and $\varepsilon_{poly}$.

Another point worth noting is that although polymers follow the general trend of inorganic compounds, they do not seem to have the high dielectric constant outliers that inorganics exhibit. We believe this is due to the fact that inorganics, being structurally more ordered than polymers, can benefit from a significant contribution to the dielectric constant from the optical phonon modes.

The discussion above provides insight on the search for new high-k dielectrics that break the inverse relationship apparent in both Figs 4 and 5. Thus, we suggest that materials with the following characteristics might have superior dielectric properties:

1. Flat conduction and valences band (d and f orbitals might help achieve this).
2. Crystal symmetries that have been known to have significant ionic contributions to the dielectric constant (e.g., Fm$\overset{¯}{3}$m, R3c).

However, we emphasize that the ionic components tend to zero at high field frequencies and hence, the effective dielectric constant might be significantly different at THz or GHz applications.

### Low-k dielectrics

Since the band gap can be thought as a proxy to how insulating a material is, good low-k dielectrics will also have large *E*_*g*_. However, in this case the advantage is that high band gap materials naturally have a low dielectric constant. Additionally, suppressing the ionic contributions might be beneficial. For this, the selection of low symmetry structures and elements with small difference in electronegativity may be helpful.

## Technical Validation

The high-throughput calculation methodology and workflow used in the present study were validated in Petousis *et al.*^4^. Specifically, the eigenvalues of the total dielectric tensor were compared to experimental values for a set of representative compounds. This set was made up of 88 compounds consisting of 42 different elements and belonging to 14 different point groups. In cases where larger than average deviations from experiments existed, the quality of the results was ensured by confirming agreement with other *state-of-the-art* and *compound-bespoke* DFPT calculations reported in the literature. In the same reference, the method for calculating the refractive index at optical frequencies and far from resonance was also validated by comparing against experimental data found in the literature for a subset of 87 compounds.

**Figure 7:**
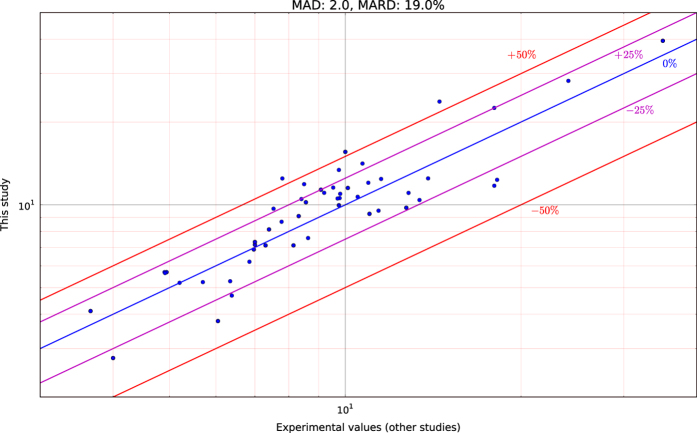
Comparison of calculated values. $\left(\varepsilon_{poly}\right)$ versus experimental values in the literature. The lines indicate the relative deviation of the calculated values with respect to experiments. The Mean Absolute Deviation (MAD) and Mean Absolute Relative Deviation (MARD) were extracted as 2.0 and 19.0% respectively. Data points are listed in Table 4.

As described in more detail in the Methods section, each calculation was tested for validity by checking the acoustic phonons at the Gamma point and the symmetry of the dielectric tensor. Furthermore, when the information was available, our results were checked against other experimental values reported in the literature. The comparison is presented in Fig. 7 and Table 4. We observe that in most cases materials deviate less than +/−25% from experiments. There are many factors that are not included in the DFPT model and contribute to this deviation e.g., (1) temperature, (2) pressure, (3) grain boundaries, (4) defects, (5) surface effects, (6) phonon anharmonicity. It should be noted that experimental values also vary between different studies. A detailed analysis of the reasons for deviation from experiments can be found in Petousis *et al.*^4^. The Mean Absolute Deviation (MAD) and Mean Absolute Relative Deviation (MARD) were 2.0 and 19.0% respectively, which we consider acceptable for a screening methodology. Once promising candidate materials are identified, further calculations and analyses can be performed to obtain a better estimate.

**Figure 8:**
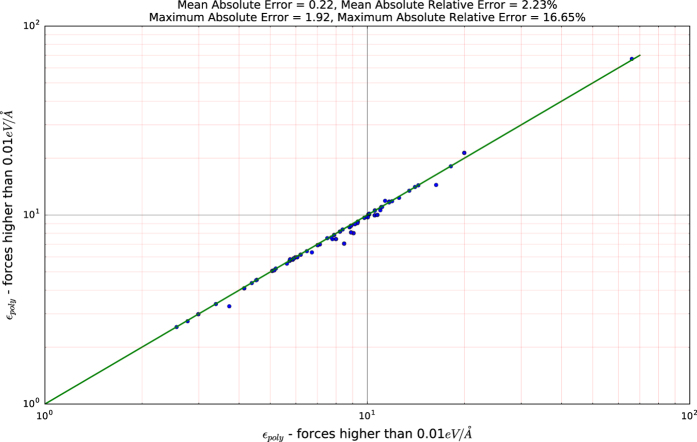
Plot showing the effect of remnant interatomic forces. The errors where calculated by taking the dielectric constant with forces less than 0.01 *eV*/Å as the ‘correct’ value.

Furthermore, Fig. 8 shows the effect of structural relaxation and remnant interatomic forces on the dielectric constant. In particular, we plot the dielectric constant for a subset of 90 compounds where on the x-axis, interatomic forces are less than 0.05 *eV*/Å but higher than 0.01 *eV*/Å and on the y-axis they are less 0.01 *eV*/Å for the same compounds. Figure 8 shows that although the deviation between the two cases, is on average relatively small (0.22 absolute and 2.23% relative deviations), there are cases for which this deviation can be significant (e.g., 1.92 and 16.65%).

## Usage Notes

We present a database of calculated dielectric constant and refractive index for 1,056 compounds. Our work should be of interest to researchers and engineers from a number of different fields, for example, electronic structure theory, photovoltaics and electronic devices. We expect this database to be used in the understanding of dielectric materials and in the search for new dielectrics with unique and tailored properties. Additionally, it can be used in the screening of replacement candidates for currently used dielectrics such as SiO_2_. The above use cases are facilitated by the Materials Project website interface which allows users to search for materials with target dielectric response or refractive index. Furthermore, the user can specify additional constraints such as stability, band gap and/or density. In line with the Materials Project practice, users will be able to request calculated dielectric constants for compounds that are not currently listed. The existence of a database such as the one presented here, opens opportunities in data intensive Materials Science. For example, the application of machine learning techniques, could lead to the identification of structural and chemical features that are key to the dielectric response. Such features would not only enhance the theoretical understanding but could also accelerate the discovery of novel dielectric materials.

## Additional information

**How to cite this article:** Petousis, I. *et al.* High-throughput screening of inorganic compounds for the discovery of novel dielectric and optical materials. *Sci. Data* 4:160134 doi: 10.1038/sdata.2016.134 (2017).

**Publisher’s note:** Springer Nature remains neutral with regard to jurisdictional claims in published maps and institutional affiliations.

## Supplementary Material



## Figures and Tables

**Table 1:** Dielectric tensors shape for different crystal symmetries.

**Crystal System**	**Point Groups**	**Dielectric Tensor**	**No. of independent elements**
Cubic	23, m$\overset{¯}{3}$, 432$\overset{¯}{4}$3m, m$\overset{¯}{3}$m	$\left(\begin{array}{ccc} \varepsilon_{11} & 0 & 0\\ 0 & \varepsilon_{11} & 0\\ 0 & 0 & \varepsilon_{11} \end{array}\right)$	1
Hexagonal, Trigonal and Tetragonal	3, $\overset{¯}{3}$, 32, 3m, $\overset{¯}{3}$m4, $\overset{¯}{4}$, 4/m, 4224mm, $\overset{¯}{4}$2m, 4/mmm6 $\overset{¯}{6}$, 6/m, 6226mm, $\overset{¯}{6}$m2, 6/mmm	$\left(\begin{array}{ccc} \varepsilon_{11} & 0 & 0\\ 0 & \varepsilon_{11} & 0\\ 0 & 0 & \varepsilon_{33} \end{array}\right)$	2
Orthorhombic	222, mm2mmm	$\left(\begin{array}{ccc} \varepsilon_{11} & 0 & 0\\ 0 & \varepsilon_{22} & 0\\ 0 & 0 & \varepsilon_{33} \end{array}\right)$	3
Monoclinic	2, m, 2/m	$\left(\begin{array}{ccc} \varepsilon_{11} & 0 & \varepsilon_{13}\\ 0 & \varepsilon_{22} & 0\\ \varepsilon_{13} & 0 & \varepsilon_{33} \end{array}\right)$	4
Triclinic	1, $\overset{¯}{1}$	$\left(\begin{array}{ccc} \varepsilon_{11} & \varepsilon_{12} & \varepsilon_{13}\\ \varepsilon_{12} & \varepsilon_{22} & \varepsilon_{23}\\ \varepsilon_{13} & \varepsilon_{23} & \varepsilon_{33} \end{array}\right)$	6

**Table 2:** Description of metadata keys.

**Key**	**Datatype**	**Description**
e_electronic	array	$\varepsilon_{ij}^{\infty }$—Dielectric tensor (electronic contribution)
e_total	array	$\varepsilon_{ij}$—Total dielectric tensor
poly_electronic	number	$\varepsilon_{poly}^{\infty }$—Polycrystalline dielectric constant estimate (electronic contribution)
poly_total	number	$\varepsilon_{poly}$—Polycrystalline dielectric constant estimate (total)
n	number	*n*—refractive index
band_gap	number	Band gap in the Materials Project database
pot_ferroelectric	boolean	if ‘True’ it signifies a potentially ferroelectric compound
meta	various	metadata—contains details about the structure and DFPT calculation

**Table 3:** Description of metadata keys.

**Key**	**Datatype**	**Description**
material_id	string	Materials Project ID number
formula	string	Chemical formula
structure	string	Crystal structure in Crystallographic Informaiton File (CIF) format
high_forces	boolean	‘True’ if remnant interatomic forces are larger than 0.01 *eV/*Å
poscar	string	Crystal structure in the VASP-specific poscar format
kpoints	string	k-points in the VASP-specific kpoints format
incar	string	simulation parameters definition file in the VASP-specific incar format
potcar	string	names of the VASP pseudopotentials used for each element
point_group	string	Point group in Hermann-Mauguin notation
space_group	number	Space group number as defined by the International Union of Crystallography
nsites	number	number of atoms in the primitive cell
kpoint_density	number	Number of k-points in the first Brillouin zone per reciprocal atom

**Table 4:** Comparison with experimental data.

**Compound**	**MP ID**	$\varepsilon_{poly}$	$\varepsilon_{poly}^{exp.}$	**Compound**	**MP ID**	$\varepsilon_{poly}$	$\varepsilon_{poly}^{exp.}$
MnF_2_	mp-560902	7.12	8.15^32^	AgI	mp-22894	7.16	7.00^33^
RbBr	mp-22867	5.69	4.90^34^	Li_3_N	mp-2251	10.69	10.50^35^
BN	mp-984	4.68	6.39^36^	GaN	mp-830	10.96	9.80^37^
BP	mp-1479	9.27	11.00^38^	AgI	mp-22925	7.32	7.00^33^
NiF_2_	mp-559798	5.20	5.20^39^	RbI	mp-22903	5.69	4.94^34^
MoS_2_	mp-2815	9.76	12.72^40^	RbCl	mp-23295	5.65	4.91^34^
CaSe	mp-1415	12.47	7.80^41^	KN_3_	mp-827	6.21	6.85^42^
ZnO	mp-1986	11.35	9.08^34^	HgS	mp-9252	12.33	18.20^43^
SiC	mp-7140	10.58	9.78^44^	ZnTe	mp-8884	11.52	10.10^43^
MoSe_2_	mp-1634	11.73	18.00^45^	ZnF_2_	mp-1873	8.14	7.40^39^
ZnS	mp-560588	9.10	8.32^46^	LaCl_3_	mp-22896	10.22	8.56^47^
Ga_2_Se_3_	mp-1340	12.03	10.95^48^	Cr_2_O_3_	mp-19399	11.05	12.83^34^
AsF_3_	mp-28027	5.24	5.70^34^	SnS_2_	mp-9984	12.48	13.86^49^
PI_3_	mp-27529	4.11	3.66^50^	BaSe	mp-1253	14.13	10.70^41^
SrSe	mp-2758	11.89	8.50^41^	GaS	mp-2507	7.57	8.63^51^
As_2_Se_3_	mp-909	10.41	13.40^52^	SiC	mp-11714	10.54	9.70^53^
InSe	mp-22691	9.68	7.53^54^	HCl	mp-632326	2.77	4.00^55^
TlF	mp-558134	39.49	35.00^56^	FeS_2_	mp-226	28.24	24.11^57^
KMgF_3_	mp-3448	6.88	6.97^58^	KBrO_3_	mp-22958	7.12	7.30^34^
KMnF_3_	mp-555123	13.39	9.75^58^	KMnF_3_	mp-555359	9.96	9.75^58^
AlCuS_2_	mp-4979	8.68	7.78^59^	Cd(GaS_2_)_2_	mp-4452	9.52	11.40^60^
ZnSiP_2_	mp-4763	12.42	11.52^60^	AlPO_4_	mp-7848	3.78	6.05^34^
GaCuS_2_	mp-5238	11.55	9.53^61^	ZnSnP_2_	mp-4175	15.58	10.00^60^
BaSnO_3_	mp-3163	22.51	18.00^34^	Cd(GaSe_2_)_2_	mp-3772	11.06	9.20^60^
NaNO_2_	mp-2964	5.27	6.35^62^	GaAgS_2_	mp-5342	10.49	8.41^60^
BiTeI	mp-22965	23.74	14.50^60^				

## References

[d1] Dryad Digital Repository Petousis I. 2017 http://dx.doi.org/10.5061/dryad.ph81h

